# Bridging the Gap Between Theory and Practice: Assessing Two Decades of Methodological Procedures and Reporting Omissions in Roadkill Studies of The Brazilian Cerrado

**DOI:** 10.1007/s00267-026-02564-4

**Published:** 2026-08-17

**Authors:** Natalia de Quadros Pereira, Carine Firmino Carvalho-Roel

**Affiliations:** 1 Laboratório de Ecologia de Mamíferos (LEMA), Instituto de Biologia, Universidade Federal de Uberlândia, Uberlândia, Brasil; 2 Laboratório de Ecologia e Conservação (LAEC), Departamento de Biologia, Faculdade de Filosofia, Ciências e Letras de Ribeirão Preto, Universidade de São Paulo (FFCLRP/USP), Ribeirão Preto, Brasil

**Keywords:** Animal mortality, Bias correction, Road Ecology, Road network, Wildlife mortality, Wildlife-vehicle collision

## Abstract

This review assesses two decades (2000–2024) of Road Ecology research in the Brazilian Cerrado, a global biodiversity hotspot. We analyzed 63 studies on four major vertebrate groups to quantify trends, methodological procedures, omissions, and road mortality, drivers that could compromise the evidence base for environmental management. While research output has expanded temporally, it remains geographically concentrated in established states (Goiás, Minas Gerais, and São Paulo), leaving high-deforestation frontiers like the Matopiba region (Tocantins, Maranhão, Piauí, and Bahia) severely under-represented. Methodologically, the prevailing reliance on high-speed vehicle surveys (48% of studies; mean 53 km/h) and the scarcity of foot-based monitoring lead to a systematic underestimation of small-bodied taxa. Furthermore, 90% of the studies failed to report at least one key methodological procedure; specifically, 51% omitted the number of observers, 49% failed to report traffic volume, and even the total distance monitored and monitoring frequency were not specified in 11% and 16% of the studies, respectively. While methodological reporting has improved over time, omissions remain influenced by bibliometric drivers; specifically, larger authorship scales and articles often constrain methodological disclosure. Observed roadkill rates (ranging from 0.008 to 0.030 ind/km/day) reflect detection and carcass persistence biases rather than ecological reality, with highly detectable mammals and birds dominating records. To bridge the gap between theory and applied science, we propose a Minimum Reporting Checklist. It ensures data reporting and comparability, providing a universal standard to enhance the technical rigor of academic research, environmental licensing, and wildlife mitigation policy globally.

## Introduction

Roads, while essential for economic development, represent a significant global challenge to biodiversity conservation and human safety by disrupting ecological processes (van der Ree et al., 2015). To better understand and reduce these conflicts, the field of Road Ecology emerged (Forman, 1998), focusing on the negative impacts of roads, including habitat fragmentation, barrier effects, and roadkill (Forman and Alexander, 1998; van der Ree et al., 2015). Roadkill ranks as the second leading cause of wildlife mortality among anthropogenic threats (Hill et al., 2019), and the need for reliable, applicable data to inform mitigation investment is a critical global priority (Grilo et al., 2025).

Beyond the direct ecological consequences, roadkill represents a significant threat to human safety and causes substantial economic loss. Collisions involving medium and large-sized animals frequently result in severe vehicle damage, human injury, and even fatalities, leading to immense costs associated with emergency response, medical care, property damage, and traffic delays (Abra et al., 2019; Huijser et al., 2009). Therefore, consistent data are necessary for transportation agencies and governments to allocate resources effectively for mitigation and accident prevention.

In megadiverse countries like Brazil, the scale of wildlife mortality due to roadkill is alarming. Initial studies estimated that more than 8 million birds and over 2 million mammals are killed each year on the country’s paved roads (González-Suárez et al., 2018). More recent projections suggest that up to 9 million medium and large-sized mammals may die annually (Pinto et al., 2022). For many endangered species, roadkill is a recognized threat (Carvalho-Roel et al., 2024; Desbiez et al., 2020; Medici and Desbiez, 2012; Grilo et al., 2021). The added value of roadkill data extends far beyond simple mortality rates. When collected extensively, these data offer insights into population dynamics, animal behavior, and environmental health (Schwartz et al., 2020). Consequently, these technical data have become core to national environmental policy; for instance, the “Plano de Redução de Impacto de Infraestruturas Viárias Terrestres sobre a Biodiversidade” (PRIM-IVT) officially recognizes road networks as a primary threat to national biodiversity and establishes guidelines for planning and regularizing terrestrial transport infrastructure with lower environmental impact (ICMBio/MMA, 2018).

Despite the severity of the problem and the immense potential of these data, the lack of standardized roadkill monitoring protocols is a pervasive global barrier that limits data comparability and the efficacy of mitigation planning (Collinson et al., 2014). Key sources of error, including carcass persistence and detection bias, lead to severe underestimations, especially of small-bodied taxa (Barrientos et al., 2018; Santos et al., 2016; Teixeira et al., 2013). Additional factors influencing roadkill include the level of surrounding urbanization (Rendall et al., 2021), the presence of Protected Areas (PAs), road surface type (Figueiredo et al., 2013; Pereira et al., 2025), road density, vehicle flow, speed (Arroyave et al., 2006; Rendall et al., 2021), highway concession status (Abra et al., 2018), and the presence of wildlife crossings (Rytwinski et al., 2016). Therefore, acknowledging heterogeneity in monitoring methods is essential to ensure data robustness and comparability, to ground conservation decisions in reliable evidence, and to effectively reduce wildlife mortality on roads. Although most of these factors are well-known as influencing roadkill rates, a global review of studies published until 2012 found that 72% (44 out of 61 assessed) provided limited or no information on fundamental procedures such as monitoring speed, number of observers, and sampling frequency or distance. (Collinson et al., 2014).

Brazil leads Latin America in Road Ecology research (Pinto et al., 2020). Previous syntheses have focused primarily on taxonomic or geographical patterns (Dornas et al., 2012; Oliveira et al., 2020), extrapolating mortality numbers across broad scales (Pinto et al., 2022), sharing data (Grilo et al., 2018), and comparing studies surveying roads with and without PAs (Miranda and Schiavetti, 2024). However, no research has yet performed a systematic diagnostic assessment of the literature to quantify the quality and consistency of methodologies employed across these studies.

Within the Brazilian context, the Cerrado domain represents a critical frontier for infrastructure expansion and conservation conflict (Myers et al., 2000). As the world’s richest tropical savanna, it has already lost over half of its original vegetation to intensive agribusiness (Strassburg et al., 2017). Federal and state networks total 110,000 km of roads, with a planned expansion of 10% (ICMBio/MMA, 2018). This rapid infrastructure growth creates an ecological trap. According to the diagnostics established by the PRIM-IVT, 53% of federal and 48% of state roads are located in highly vulnerable ecological areas (ICMBio/MMA, 2018). Consequently, qualifying the robustness of existing roadkill monitoring protocols is a critical regulatory priority; without standardized data, future infrastructure licensing may underestimate wildlife mortality and fail to implement effective mitigation measures along these expanding corridors.

This study performs a systematic review of two decades of Road Ecology research in the Brazilian Cerrado to quantify the consistency of survey procedures and identify the underlying drivers of data omission. While we focus on the Cerrado as a critical case study, the methodological inconsistencies identified here reflect systemic challenges facing the global Road Ecology community. By building on previous evidence of high global omission rates (Collinson et al., 2014), our analysis assesses whether these trends persist in modern literature and provides a scalable diagnostic framework applicable to roadkill monitoring programs worldwide. Based on our findings of data omission, we propose a Minimum Reporting Checklist (MRC). By identifying which specific parameters are most frequently neglected (e.g., traffic volume, road stretch length, or carcass persistence trials), this MRC serves as a bridge between academic research and standardized monitoring required for environmental licensing and more robust analyses. Our specific objectives are: (a) to quantify the temporal and spatial trends in research output, including author characteristics and publication type; (b) to systematically assess and quantify the methodological heterogeneity and reporting completeness of roadkill surveys, focusing on sampling effort, survey parameters (e.g., vehicle speed, observer number), and road management metrics (e.g., traffic volume, concession status); (c) to identify the bibliometric parameters (e.g., authorship scale, publication format) that influence methodological omissions; (d) to synthesize roadkill patterns across major vertebrate groups to identify potential biases associated with current survey designs. Ultimately, this study evaluates the extent to which decades of theoretical Road Ecology knowledge have been successfully translated into practice, providing a roadmap for more transparent and comparable data reporting in the future.

## Materials and Methods

### Literature Search and Selection Procedure

We conducted a systematic literature search for peer-reviewed studies and gray literature (including doctoral dissertations, master’s theses, and conference proceedings) published between 2000 and 2024. We used the search engine Google Scholar and the online database CAPES Journal Portal (“Portal de Periódicos da CAPES”), a key Brazilian virtual library providing access to scientific publications and regional gray literature (https://www-periodicos-capes-gov-br.ez34.periodicos.capes.gov.br/index.php). In CAPES Journal Portal, we utilized the integrated search tool, which simultaneously queries major indexed databases, including Web of Science, Scopus, and ScELO. The keywords used were (“atropelamento de fauna” OR “atropelamento de vertebrado” OR “atropelamento” OR “fauna atropelada”) AND “Cerrado”, and their English equivalents: (“wildlife roadkill” OR “vertebrate roadkill” OR “roadkill” OR “wildlife-vehicle collision” OR “vertebrate mortality”) AND (“Cerrado” OR “Savannah”). Relevant literature was also searched for in bibliographies of literature reviews, such as those mentioned in the text.

Studies identified through the search were evaluated for inclusion at three successive levels to minimize selection bias: (i) title screening: all records were initially screened by title by a single reviewer; in cases of uncertainty, the reviewer opted for inclusion; (ii) abstract screening: each potentially relevant study was then judged based on its abstract. Again, a conservative approach was taken where uncertainty resulted in inclusion for full-text analysis; (iii) full-text review: finally, the remaining studies were subjected to a rigorous full-text review to confirm eligibility based on the following criteria. We only included studies conducted specifically within the Cerrado domain that aimed at recording wildlife roadkill events and that reported both total species richness and abundance per vertebrate group. Studies were excluded if they reported roadkill rates for only one species or contained duplicated datasets.

### Data Extraction, Processing and Categorization

To synthesize and compare Road Ecology data across studies, we extracted and standardized key information. All data tables from the studies included were carefully reviewed to ensure accuracy. Unidentified individuals lacking taxonomic classification and domestic animals were excluded from the analysis.

To estimate the number of species road-killed, we applied two criteria for individuals identified only at the genus level: (i) if the genus included already-identified species in the same study, the unidentified individuals were excluded to avoid redundancy; (ii) if the genus contained only unidentified individuals, a single species was counted regardless of the number of occurrences, to prevent duplicate counting of a potentially single species. To calculate the average number of road-killed species per study and enable comparisons of species abundance and richness, we included only studies that reported data for all four major taxonomic groups: amphibians, reptiles, birds, and mammals. We extracted the most frequently road-killed species from each study’s main text; thus, more than one species could be identified as the most road-killed within the same study in cases of tied values or multiple taxonomic groups.

In studies where the roadkill rate or total distance monitored was absent or not explicitly provided, these values were inferred using available methodological information. The total distance monitored was estimated by multiplying the length of the monitored road section by the number of surveys or the sampling duration. The roadkill rate was calculated as the number of animals recorded divided by the total surveyed effort (km), using the standard metric of individuals per kilometer per day (individuals^.^km^–1.^day^–1^, hereafter ind/km/day) (Pinto et al., 2022). No correction factors for detection probability or carcass persistence were applied to standardize the roadkill rates, though we acknowledge that roadkill rates are underestimated when these factors are not considered (Barrientos et al., 2018; Santos et al., 2016; Teixeira et al., 2013). This decision was made due to methodological heterogeneity and the widespread omission of required primary data across the reviewed studies. Therefore, the resulting metrics presented herein represent observational (raw) roadkill rates.

For statistical analysis, we grouped or categorized the data to enhance comparability: (i) monitoring frequency was categorized as: monthly, surveys that covered the highway once a month; bimonthly, two to three times per month; weekly, four times per month; daily, more than once per week. In studies using two different monitoring patterns, we calculated the average frequency (total number of visits divided by study duration in months) to assign the study to a single category; (ii) for vehicle speed, if a range or multiple segments with different speeds were reported, we used the average; (iii) vehicle traffic volume was extracted directly from the primary studies. Due to the lack of standardized reporting across the literature, traffic was recorded using the available qualitative descriptors provided by the authors (e.g., “high”, “low to moderate”, “constant”, or “variable”), or as numerical values, where available, or not reported, in cases where this information was not provided; (iv) the number of lanes was grouped into: two-lanes, if the majority of the roads monitored were two-lane; four-lanes, if most of the stretches were four-lane; and mixed configurations, if the proportion of two and four-lanes was similar. When information was not available, it was classified as “not reported.”

The spatial distribution of the reviewed studies was visualized using a heatmap to identify areas of research concentration. To generate this map, the geographic coordinates (latitude and longitude) were extracted for the central point of the monitored road segment. A continuous surface of study concentration was created using Kernel Density Estimation (KDE). To identify spatial mismatches between biodiversity vulnerability to roads and roadkill research effort, we integrated the ecological sensitivity map produced by the PRIM-IVT protocol with our heatmap. The PRIM-IVT map quantifies the vulnerability of fauna, flora, and unique environments to roads by integrating key ecological attributes, including species conservation status, endemism levels, representativeness within PAs, and sensitivity to habitat fragmentation and roadkill. We performed a spatial union between both layers to generate a research priority map. Within this map, higher scores represent critical knowledge gaps, defined as regions of maximum ecological vulnerability receiving minimal to no scientific monitoring, whereas lower scores indicate either low-priority zones or areas of high sampling redundancy. This analysis was performed using QGIS 3.28.

### Statistical Analyses

We present the results as percentages to provide a general interpretation of observed trends. The Chi-square test was used to assess differences in the number of studies across various categorical methodological and contextual variables, including: number of authors, type of publication, monitoring frequency, type of vehicle used for monitoring, number of observers, vehicle traffic volume, road surface type (paved or unpaved), road type (number of lanes), administrative level responsible for the road (municipal, state, federal), highway concession status, presence of wildlife crossings, and proximity to PAs (as reported in the study) (A detailed description and definition of the methodological variables used in this assessment is provided in Table S4 of the Supplementary Material). When a significant overall effect was detected (*p* < 0.05), pairwise tests of independence for nominal data were employed to identify which specific groups differed (Mangiafico, 2018). The same test was also used to assess whether the average data collection duration (months) varied by type of publication.

To identify the factors influencing the omission of methodological information, we employed Generalized Linear Models (GLM) with a Binomial distribution (logit link function). The response variable was defined as the reporting status (0 = reported; 1 = omitted) for each methodological variable individually. We assessed nine methodological variables (omission > 10%), related to sampling effort (number of visits, total distance monitored, monitoring frequency), infrastructure characteristics (traffic volume, road lane configuration, concession status), and survey logistics (transportation method, vehicle speed, number of observers). The predictor variables included the year of publication, number of authors, publication type (Article, Proceedings, or Academic), and language (English or Portuguese). To evaluate the overall reporting quality, we calculated a total omission score for each study, defined as the sum of omissions across 12 methodological variables (Table S4). These count data wwere analyzed using a GLM with a Poisson distribution (log link function). We checked for overdispersion by comparing the residual deviance against the degrees of freedom; as no overdispersion was detected (dispersion parameter ≈ 1), the standard Poisson model was retained. Multicollinearity was assessed using Generalized Variance Inflation Factors (GVIF). All variables showed GVIF^(1/(2Df)) values below the threshold of 2.5, indicating no severe collinearity issues (Fox and Monette, 1992). The significance of each predictor was assessed using the Likelihood Ratio Test (LRT), with results reported as Chi-square (χ²) and degrees of freedom (df). Significant differences between types of publication were identified through pairwise post-hoc comparisons using the “glht()” function in the “multcomp” package (Hothorn et al., 2002).

To evaluate differences in the number of road-killed species, number of individuals (abundance), and raw roadkill rate across the four major vertebrate groups, we applied the Kruskal-Wallis test due to the non-parametric nature of the data. When a significant effect was found, Dunn’s test was used as a post hoc analysis (Dinno, 2024), with p-value adjustment for multiple comparisons using the Benjamini-Hochberg method (Benjamini and Hochberg, 1995). All statistical analyses were performed using R software, version 4.5.2 (R Core Team, 2025).

## Results

Out of the 108 studies identified after abstract screening, 63 (58%) met the inclusion criteria for the final analysis (Table S1), while 45 (42%) were excluded (Table S2). The primary reasons for study exclusion were the identification of duplicated data (38%) and studies in which the focus was not related to reporting roadkill (22%).

### State‑of‑the‑Art

**Fig. 1:**
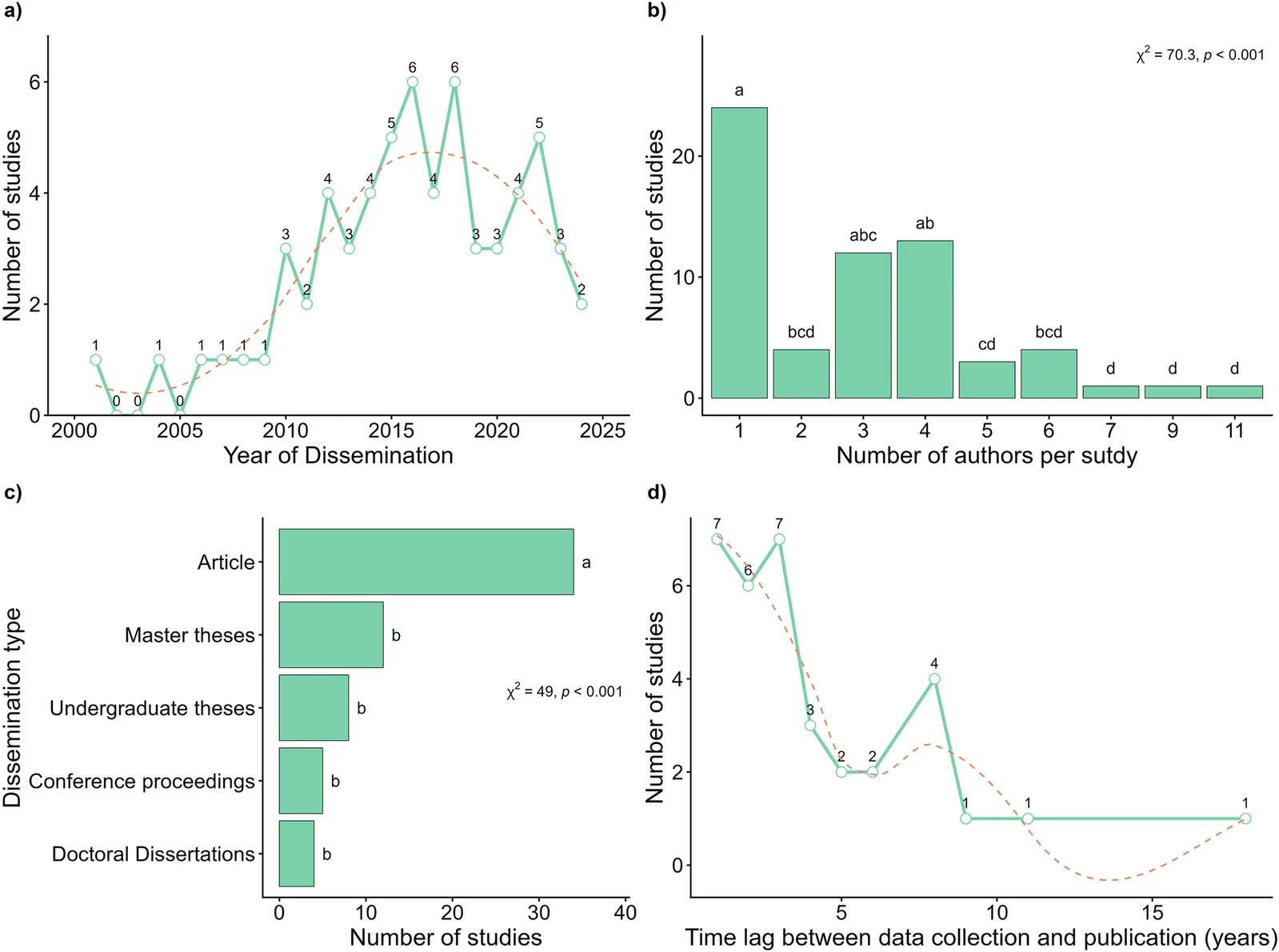
State-of-the-art of two decades of roadkill studies in the Brazilian Cerrado (2000-2024). **a** Number of studies per year; **b** Number of studies by number of authors; **c** Number of studies by dissemination format; **d** Interval between data collection completion and publication, in years. Different letters indicate statistically significant differences (*p* < 0.05); groups sharing the same letter do not differ significantly from one another, whereas groups with distinct letters are statistically different

The number of studies exhibited a clear increase over the past two decades. Study output remained low until 2009, followed by a rise that peaked in 2018 (Fig. 1a). A subsequent decline was observed after 2018 (*n* = 20 from 2019 to 2024), relative to the preceding six-year period (*n* = 28 from 2014 to 2018). The mean annual number of studies was 2.7 ( ± 1.9), with a maximum of 6 studies disseminated in a single year.

A significant difference in the number of authors per study was detected (χ² = 70.3, df = 8, *p* < 0.001). A great part of the studies (38%, *n* = 24) were conducted by a single author, typically as part of academic requirements (e.g., dissertations or theses) (Fig. 1b).

The type of dissemination also varied significantly (χ² = 49, df = 4, *p* < 0.001). Articles were the most common format, accounting for 54% (*n* = 34) of the studies (Fig. 1c). The mean time between the end of data collection and journal publication for the 34 articles was 4 years (± 3.9), with a range of 1 to 18 years (Fig. 1d). Articles were published across 29 different scientific journals (Table S3). Most studies (78%) were written in Portuguese.

**Fig. 2:**
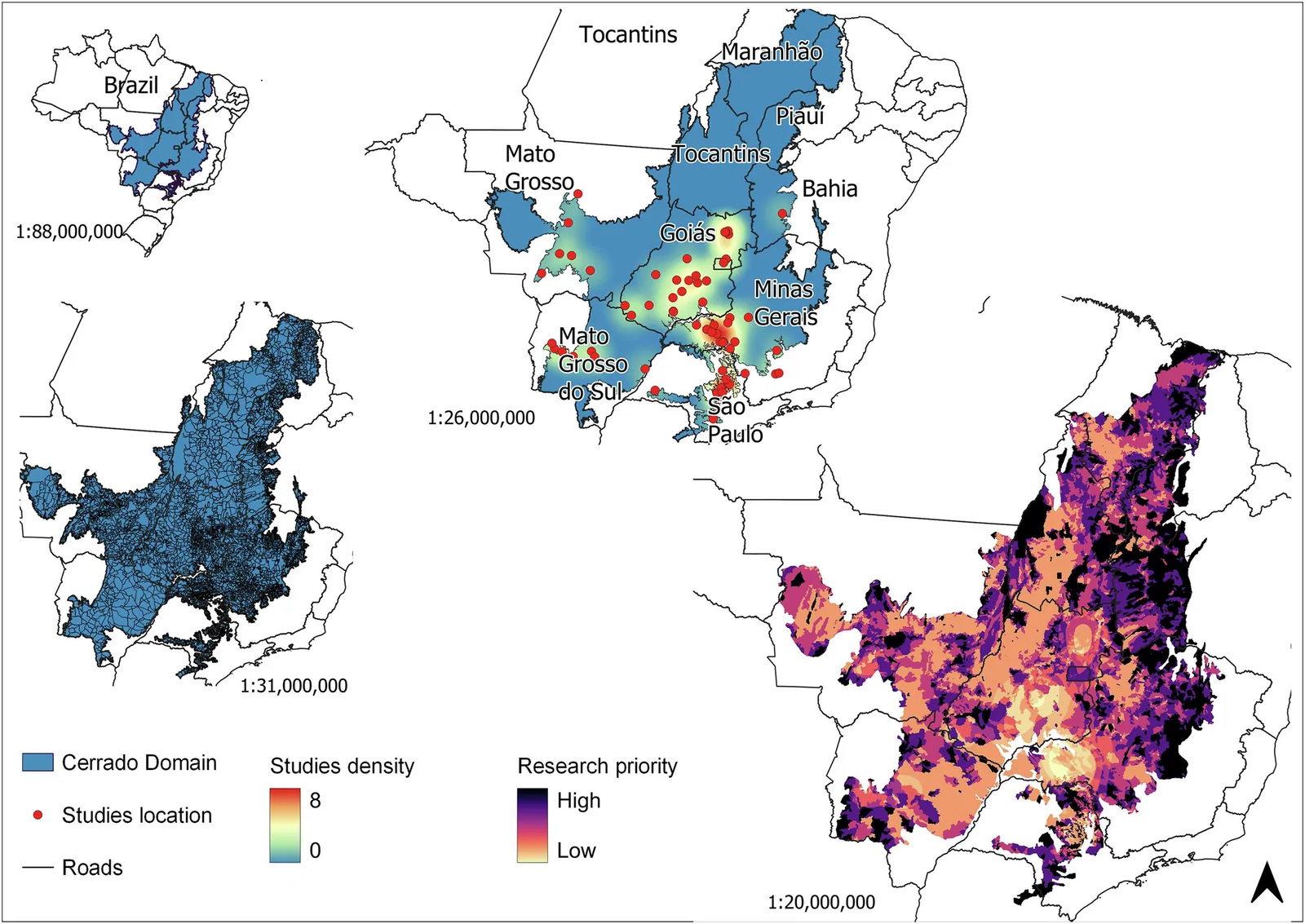
Spatial distribution of roadkill research effort and research priorities in the Cerrado biome (2000–2024). The continuous gradient (heatmap, top right) displays historical research concentration via Kernel Density Estimation (KDE). Overlapping polygons represent the research priority (bottom right), derived from the union of research effort and the PRIM-IVT ecological sensitivity map (ICMBio/MMA, 2018). Highest scores indicate critical knowledge gaps where maximum biodiversity vulnerability overlaps with minimal to no scientific monitoring. Brazilian states and federal, state, and municipal road networks are shown for geographic reference

Studies were disproportionately concentrated within the Cerrado biome (Fig. 2). While most research was concentrated in the states of Goiás (35%, *n* = 23), Minas Gerais (30%, *n* = 20), and São Paulo (15%, *n* = 10), no studies were recorded from the states of Tocantins, Maranhão, or Piauí. Regions of high ecological vulnerability to roads receiving minimal to no scientific monitoring are concentrated in the northern and eastern regions of the biome, particularly within the Matopiba agricultural frontier (Maranhão, Tocantins, Piauí, and Bahia states) and ecotonal zones. Conversely, central and southeastern regions exhibited low priority scores, indicating high sampling redundancy and low vulnerability.

### Methodological Procedures

**Fig. 3:**
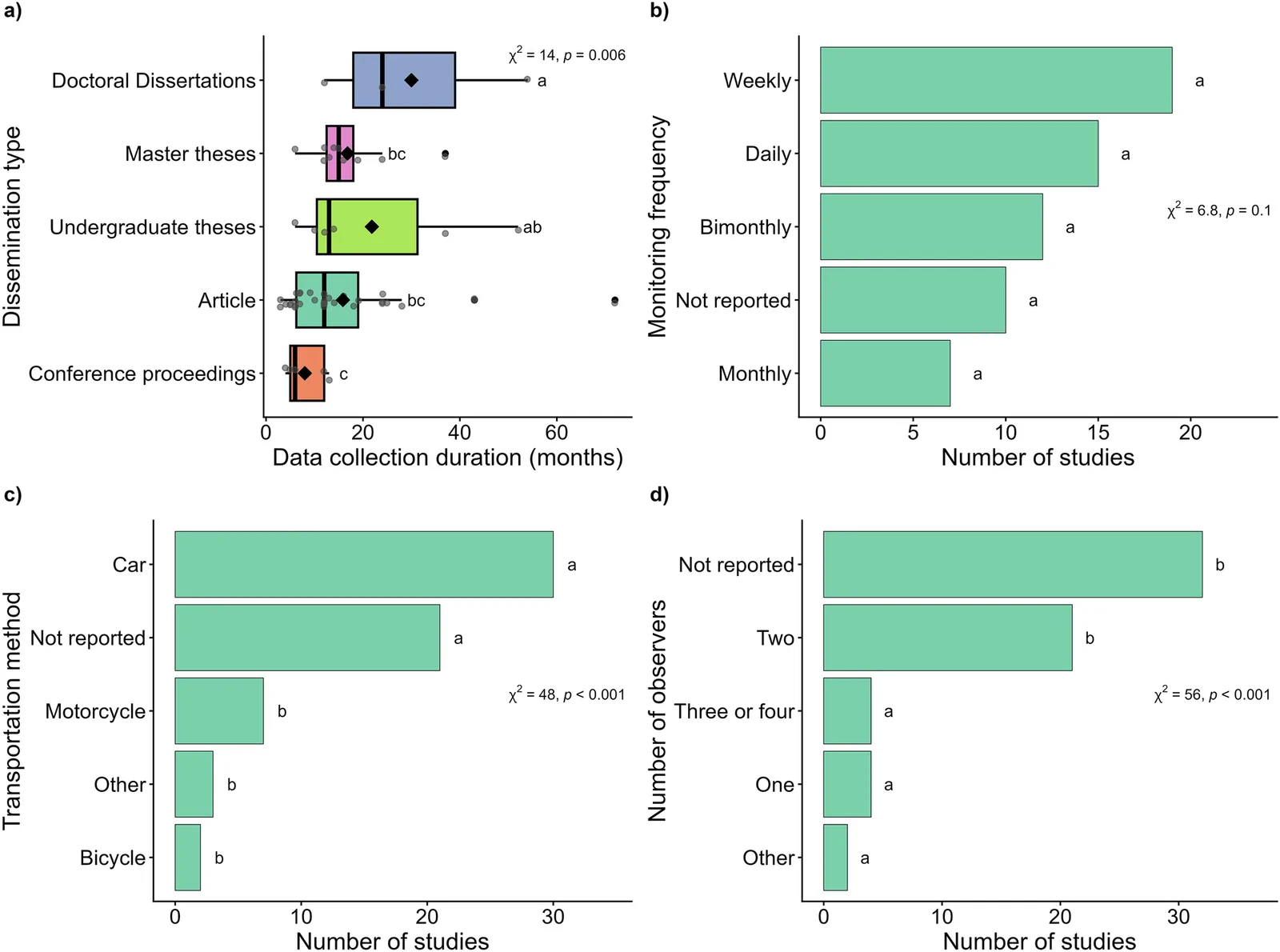
Field methodology and survey effort of roadkill studies in the Brazilian Cerrado (2000-2024). **a** Data collection duration (months) across dissemination type; **b** Frequency of monitoring applied in the surveys; **c** Transportation method used during road surveys; **d** Observer team size. Different letters indicate statistically significant differences (*p* < 0.05); groups sharing the same letter do not differ significantly from one another, whereas groups with distinct letters are statistically different

The mean duration of data collection was 16.4 months (± 13.7), ranging from 3 to 72 months. Mean collection duration was significantly shorter in conference papers (χ² = 14, df = 4, *p* = 0.006), averaging 8 months (± 4.18), and longest in doctoral dissertations, at 30 months (± 21.6) (Fig. 3a).

The mean total distance traveled per study was 8194.4 km (± 10,520), ranging from 280 km to 50,000 km (Fig. S1). Surveys monitored a mean stretch of 164.3 km (± 217.1), visiting it an average of 63.4 times (± 45.1) (Fig. S1). Monitoring frequency did not vary significantly (χ² = 6.8, df = 4, *p* = 0.1) (Fig. 3b).

A car was the predominant means of transportation (48%, *n* = 30) (χ² = 48, df = 4, *p* < 0.001) (Fig. 3c). Only three studies surveyed some stretches of the road on foot. The mean monitoring speed was 53 km/h (± 13.4) (Fig. S1). Half of the studies (51%, *n* = 32) did not specify the number of observers, a proportion statistically equivalent to those reporting monitoring by two people (33%, *n* = 21) (χ² = 56, df = 4, *p* < 0.001) (Fig. 3d).

**Fig. 4:**
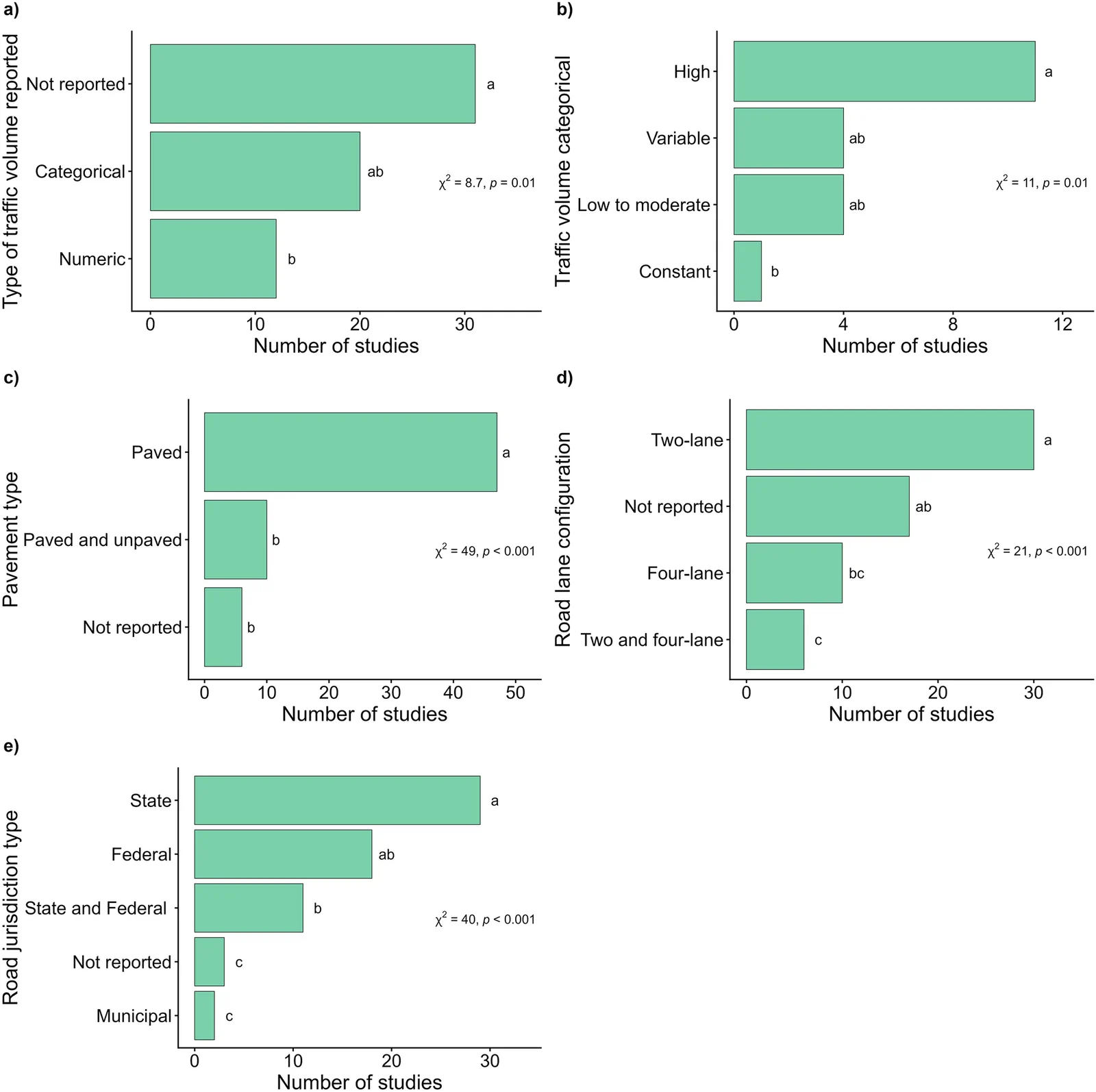
Road characteristics where roadkill studies were performed in the Brazilian Cerrado (2000-2024). **a** Traffic volume data type (categorical vs numeric reporting); **b** Traffic volume categorical classification; **c** Road pavement type; **d** Road lane configuration; **e** Road jurisdiction type. Different letters indicate statistically significant differences (*p* < 0.05), groups sharing the same letter do not differ significantly from one another, whereas groups with distinct letters are statistically different

Traffic volume varied significantly (χ² = 8.7, df = 2, *p* = 0.01), with 49% (*n* = 31) not reporting this information (Fig. 4a). Among those providing categorical descriptions, 55% (*n* = 11) classified traffic as high or intense (χ² = 11, df = 3, *p* = 0.01) (Fig. 4b).

Most studies (75%, *n* = 47) were conducted on paved roads (χ² = 49, df = 2, *p* < 0.001), with no study performed exclusively on unpaved roads (Fig. 4c). Roads with two lanes (48%, *n* = 30) were the most common (χ² = 21, df = 3, *p* < 0.001) (Fig. 4d), and most were under state jurisdiction (46%, *n* = 29) (χ² = 40, df = 4, *p* < 0.001) (Fig. 4e).

In terms of concession status, 51% (*n* = 32) of studies did not report whether the road was under concession. Proximity to PAs did not vary significantly (χ² = 0.78, df = 1, *p* = 0.4), with 44% (*n* = 28) of studies conducted near such areas. Notably, only five studies (8%) were performed on roads equipped with wildlife crossings (χ² = 46, df = 1, *p* < 0.001).

### Methodological Omission

**Fig. 5:**
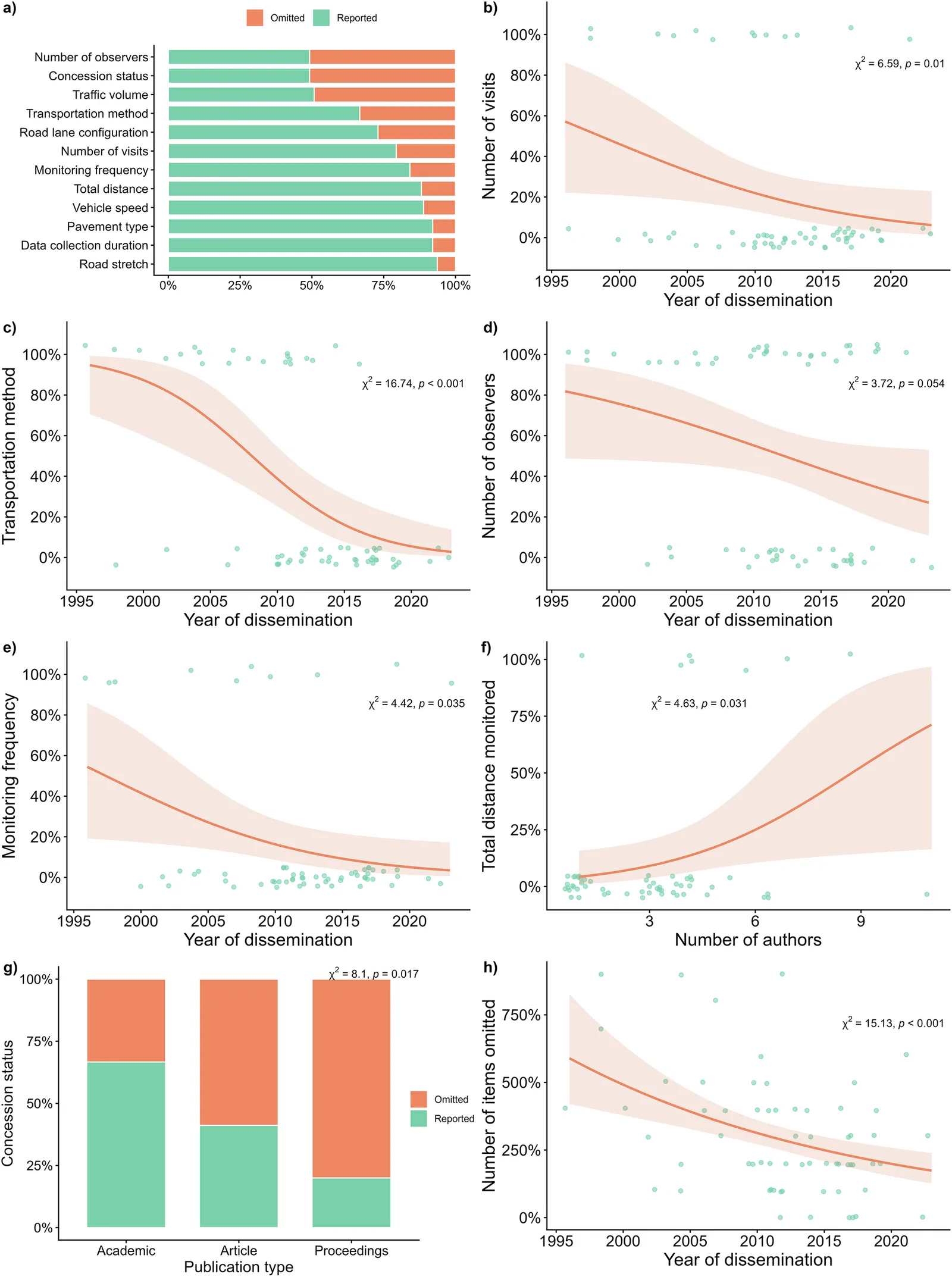
Patterns and drivers of methodological omission in roadkill studies conducted in the Brazilian Cerrado (2000-2024). **a** Proportion of reported (green) and omitted (orange) data for 12 methodological variables across all reviewed studies; Omission of information about the: **b** number of visits, **c** transportation method, **d** number of observers, and **e** monitoring frequency over the years; **f** Omission of total distance monitored by the number of authors; **g** Reporting of road concession status by publication type; **h** Total number of omitted methodological items per study over time. Shaded areas represent the 95% confidence intervals. Likelihood Ratio Test results (χ²) and p-values are indicated for each model

Methodological data omission varied across the assessed methodological variables. The most consistently reported parameters (lowest omission) were road stretch length (6%, *n* = 4), data collection duration, and pavement type (8%, *n* = 5, each). In contrast, the most frequently omitted variables were traffic volume (49%, *n* = 31), concession status, and the number of observers (51%, *n* = 32, each) (Fig. 5a). Notably, even critical parameters such as total distance monitored were not explicitly reported in 21% (*n* = 13) of the studies, though this could be inferred or calculated for six of them.

Temporal trends significantly influenced reporting quality. The year of publication was negatively associated with the omission of the number of visits (χ² = 6.59, df =1, *p* = 0.010), transportation method (χ² = 16.74, df =1, *p* < 0.001), number of observers (χ² = 3.72, df =1, *p* = 0.054), and monitoring frequency (χ² = 4.42, df =1, *p* = 0.035) (Fig. 5b–e; Table S5). Conversely, studies with larger author affiliations omitted the total distance traveled more frequently (χ² = 4.63, df =1, *p* = 0.031) (Fig. 5f). Academic publications were more likely to report road concession status than other publication types (χ² = 8.1, df =2, *p* = 0.017) (Fig. 5g). Overall, the total omission score significantly decreased over time (χ² = 15.13, df =1, *p*  <0.001), indicating an improvement in methodological reporting (Fig. 5h).

### Roadkill Patterns: Richness, Abundance and Rates

**Fig. 6:**
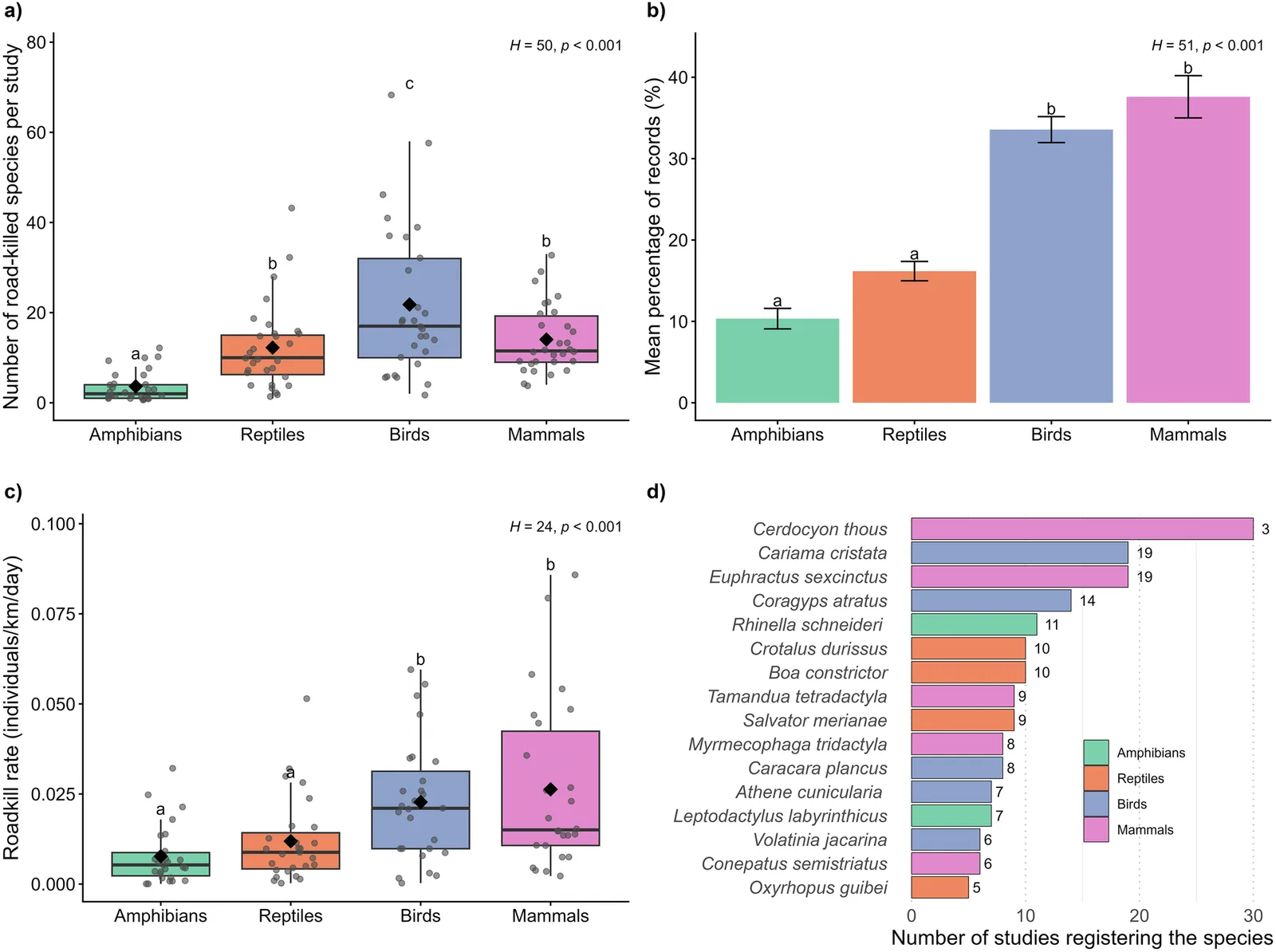
Roadkill patterns: richness, abundance, rates, and most road-killed species across the four major vertebrate groups (Amphibians, Reptiles, Birds, and Mammals) during two decades of roadkill studies in the Brazilian Cerrado (2000-2024). **a** Number of road-killed species per study; **b** Mean percentage of records of vertebrates’ roadkill; **c** Roadkill rates (individuals/km^/^day, raw data); **d** Frequency of citation (number of studies) identifying each species as the most frequently road-killed (the total sum exceeds the number of reviewed studies as more than one species could be identified as the most road-killed within the same study). Different letters indicate statistically significant differences (*p* < 0.05); groups sharing the same letter do not differ significantly from one another, whereas groups with distinct letters are statistically different

The number of road-killed species varied significantly across vertebrate groups (H = 50, df = 3, *p* < 0.001). Birds exhibited the highest diversity, with a mean of 23.9 species (± 20.1) per study (Fig. 6a). Mammals and reptiles followed, with means of 14 species (± 7.6) and 12.2 species (± 9.5), respectively. Amphibians showed the lowest diversity, with a mean of 3.6 species (± 3.2). Overall, studies reported a mean of 53.9 ( ± 34.8) species.

Abundance of roadkill records also varied significantly (H = 51, df = 3, *p* < 0.001). Mammals and birds were the most recorded groups, accounting for 38% and 34% of all records, respectively (Fig. 6b). Amphibians and reptiles were the least represented, totaling 11% and 16% of the records, and did not differ significantly from each other.

Raw roadkill rates were available or calculated for 53 studies (84% of those analyzed), but only 27 (43%) assessed the four vertebrate groups. A significant difference was observed among roadkill rates for vertebrate groups (H = 24, df = 3, *p* < 0.001). Amphibians and reptiles exhibited the lowest collision rates and did not differ statistically, averaging 0.008 ( ± 0.008) and 0.012 ( ± 0.012) ind/km/day, respectively (Fig. 6c). Birds and mammals showed the highest collision rates and did not differ from each other, averaging 0.023 ( ± 0.017) and 0.030 ( ± 0.030) ind/km/day, respectively. The overall roadkill rate across all groups was 0.073 ( ± 0.046) ind/km/day.

The species most frequently cited as the most road-killed were: (i) Amphibians: *Rhinella schneideri* (cururu toad) in 11 studies and *Leptodactylus labyrinthicus* (pepper frog) in 7 studies (Fig. 6d); (ii) Reptiles: *Crotalus durissus* (rattlesnake) and *Boa constrictor* (boa) in 10 studies each, and *Salvator merianae* (teiú) in 9 studies; (iii) Birds: *Cariama cristata* (seriema) in 19 studies and *Coragyps atratus* (black vulture) in 14 studies; (iv) Mammals: *Cerdocyon thous* (crab-eating fox) in 30 studies and *Euphractus sexcinctus* (six-banded armadillo) in 19 studies.

## Discussion

This systematic review of roadkill studies in the Cerrado domain reveals a rapidly maturing, yet methodologically inconsistent, field in Brazil. We confirm the expansion of Road Ecology research over the last two decades. However, this scientific growth is geographically concentrated in socio-economically established states (Minas Gerais, Goiás, and São Paulo), highlighting a critical knowledge gap in highly ecologically vulnerable and rapidly expanding agricultural frontiers, such as the Matopiba region (Tocantins, Maranhão, Piauí, and Bahia).

Methodologically, even with previous studies showing the importance of methodological variables to estimate roadkill rates (Collinson et al., 2014; Santos et al., 2016; Barrientos et al., 2018; Henry et al., 2021), this field suffers from inconsistency and data omission, with 90% of the studies failing to report some methodological procedure, rendering some findings non-comparable. These omissions are not random; while the overall reporting quality has significantly improved over time, specific gaps persist. We found that larger number of authors were unexpectedly associated with a higher omission of total distance monitored. Furthermore, academic publications showed better reporting of infrastructure data, such as road concession status.

This lack of standardization results in a systematic taxonomic detection bias: while birds and mammals exhibited the highest raw roadkill rates, amphibians and reptiles, despite their high global richness, showed the lowest rates. This discrepancy strongly suggests that the widespread use of inadequate protocols (i.e., vehicle-based monitoring and low frequency) leads to a systemic underestimation of roadkill rates for small-bodied taxa, compromising the accuracy of conservation planning.

While previous studies of roadkill in Brazil have provided valuable insights into taxonomic dominance and collision rates (Dornas 2012; Oliveira et al., 2020; Pinto et al., 2022) or the importance of considering carcass persistency and detectability (Santos et al., 2016; Teixeira et al., 2013), our research shifts the focus to address the question of data robustness and replicability. Our research is the first to quantify the specific methodological omissions that have historically produced inaccurate ecological inference and suggests a Minimum Reporting Checklist for future research. This unique focus allows us to move beyond simply reporting collision numbers to proposing concrete and applicable management standards.

### State‑of‑the‑Art

The observed expansion in the number of Road Ecology studies over the last two decades supports the growth trend previously reported in Brazil (Oliveira et al., 2020; Miranda and Schiavetti, 2024) and worldwide (Sukhontapatipak et al., 2025). This increase is likely associated with the institutionalization of the field in Brazil, evidenced by milestones such as the creation of the Brazilian Center for Studies in Road Ecology (CBEE) in 2012, the publication of the book “Ecologia de Estradas – Tendências e Perspectivas,” (Road Ecology - Trends and Perspectives) and the establishment of the Brazilian Network of Transportation Ecology Specialists (REET) in 2019 (https://reetbrasil.wixsite.com/reetbrasil), which helped consolidate and organize Brazilian researchers. Conversely, the decline in publication output after 2018 may be attributed to the interruption of field activities due to the COVID-19 pandemic. For example, 59.4% of geography postgraduate students in Brazil fully suspended field activities during the first 12 months of the pandemic, undoubtedly impacting monitoring data collection and, consequently, the number of subsequent publications (Silveira and Bastos, 2021). This reduction of scientific production has been noticed worldwide since 2021 (FAPESP, 2024).

Regarding dissemination format, nearly half of the primary roadkill data remains stored in university repositories, and most studies are in Portuguese. However, an even larger and more substantial fraction of primary data is contained within environmental licensing reports held by government environmental agencies. This critically restricts access and reuse of information by the broader scientific community. Despite the public disclosure requirements established by Federal Law No. 10,650/2003 and the Brazilian Access to Information Law (Law No. 12,527/2011), a structured, open-access database tailored for researchers and environmental analysts remains absent. To address this, the Brazilian Wildlife Crossing Bill, formerly known as Bill No. 466/2015, and currently under consideration in the Federal Senate as Bill No. 2550/2026, explicitly provides for the creation of such a national database. Integrating these efforts into a unified, public-access platform, such as the Brazilian Biodiversity Information System (SiBBr), is urgent to bridge this gap and optimize transport management. Centralizing these licensing and academic data into an open-access repository would not only enhance regulatory transparency but also provide baseline metadata crucial for evaluating infrastructure impacts at regional scales.

The concentration of scientific output in the states of Minas Gerais, Goiás, and São Paulo reflects the country’s socio-economic and academic context. Minas Gerais and São Paulo are the Brazilian states with the highest number of article publications (FAPESP, 2021), higher education institutions (Instituto SEMESP, 2025), and host five of the nation’s top 10 universities (CWUR, 2025), and hold the largest shares of the national Gross Domestic Product (IBGE, 2025). This higher institutional capacity and research funding directly translate into a higher volume of studies.

Despite the general advancement in scientific production, the absence of studies in the states of Tocantins, Maranhão, and Piauí, which form the Matopiba region, reveals a critical research gap. These neglected areas, along with the ecotonal zones of the northern Cerrado and the western borders near the Pantanal, are top-priority zones for urgent field monitoring. These specific high-priority sectors encompass unique ecosystems under severe threat (ICMBio/MMA, 2018). More than half of all deforested areas in Brazil in 2023 occurred in Cerrado, primarily within the Matopiba region (MapBiomas, 2024). This expansion of deforestation is likely to drastically intensify environmental impacts, including increased wildlife road mortality. For example, roadkill rates increased 144% in highway sections affected by fires within the Atlantic Forest, suggesting a direct link between deforestation, burn events, and road mortality (Lacet et al., 2023). Researchers must break the logistical convenience of roadkill sampling, shifting the research focus away from university-adjacent highways toward remote, ecologically vulnerable frontiers that lack any scientific baseline.

### Methodological Procedures

The substantial variability in monitoring duration, distance monitored, and visit frequency reflects diverse scopes and logistical constraints across the Cerrado domain studies. However, the prevailing reliance on vehicle-based monitoring as the most common method, with only three studies incorporating foot surveys, is a major source of systematic bias. High-speed monitoring by vehicle is known to reduce the detection probability of smaller carcasses, leading to the underestimation of roadkill rates for small-bodied taxa (Barrientos et al., 2018; Santos et al., 2016). Foot searches yield a substantially higher mean detection rate (e.g., 4.60 animals per stretch) compared to vehicle monitoring (e.g., 0.49 animals per stretch) (Teixeira et al., 2013). We recommend that future roadkill monitoring protocols require the integration of both methods to maximize data quality and provide a more accurate representation of mortality across all vertebrate groups.

The observed mean monitoring frequency, with 72% of studies conducting visits weekly or less frequently, represents a methodological issue because this interval is significantly longer than the average carcass persistence time, estimated at approximately less than one day to 4.93 days depending on the group (Barrientos et al., 2018; Henry et al., 2021; Santos et al., 2016; Teixeira et al., 2013). Carcass persistence directly influences detection, as intervals of two to four days reduce the probability of detection by 50% for many groups (Henry et al., 2021). To overcome the inherent bias caused by carcass removal, future research must incorporate correction factors derived from species and context-specific trials for carcass persistence. If not possible, more reliable roadkill rates can be estimated through correction factors already available in the literature. Reporting raw data alongside corrected rates is essential for true data comparability (Santos et al., 2016).

Our findings reveal knowledge gaps regarding road infrastructure and the management context. Research focused on paved, two-lane roads under state jurisdiction, with a complete absence of studies exclusively on unpaved roads. The scarcity of research on different road types limits our ability to understand the full spectrum of road impacts. Finally, the fact that only five studies were performed on roads equipped with wildlife crossings, despite the presence of over 1400 structures nationwide (DNIT, 2022) (although many of these structures may be located outside the Cerrado domain), represents a research and management gap. Without monitoring data from these structures, their effectiveness cannot be rigorously evaluated, leaving a critical failure point in conservation policy.

### Methodological Omission

While we observed a significant improvement in methodological reporting over the last two decades, our results confirm that a large proportion of studies continue to provide limited or no information on fundamental procedures, echoing global gaps identified by Collinson et al. (2014) more than 15 years ago. The failure to report fundamental metrics, such as traffic volume, concession status, and the number of observers, renders a significant portion of the roadkill data non-comparable. Specifically, omitting the number of observers, a critical proxy for detection precision and efficiency, precludes a robust assessment of sampling quality (Collinson et al., 2014). Similarly, the frequent omission of traffic volume limits the comparability of these studies, as traffic is a well-known driver of both road mortality and behavioral disturbances (Arroyave et al., 2006; Rendall et al., 2021). This omission likely stems from a lack of direct researcher access to traffic databases managed by state or federal agencies, leading to a “data-of-convenience” approach where only easily accessible variables are reported. We recommend that, where external data on traffic volume is unavailable, researchers implement simple, standardized vehicle counts to ensure the inclusion of this parameter. Furthermore, information on concession status was also missing in most studies, despite concessioned highways accounting for 25.2% of Brazil’s paved network (CNT: SEST SENAT: IT 2024). Integrating the concession element is vital for understanding the influence of private management on road safety and mitigation, although data quality caution is warranted (Abra et al., 2018).

Notably, the failure of studies to report even basic information, such as total kilometers surveyed, mean duration of data collection, and monitoring frequency, constitutes a critical omission that could impact the estimation of roadkill rates. Total surveyed distance is the fundamental metric required to standardize sampling effort and derive accurate collision rates, which are essential for comparing risk and prioritizing mitigation investments. Future studies must rigorously report total kilometers surveyed and, crucially, employ species accumulation curves to objectively validate sampling sufficiency and ensure reliable evidence for conservation planning (Collinson et al., 2014).

Our analysis shows that methodological omissions are influenced by specific bibliometric drivers. We identified an improvement in methodological procedures reporting over time, with recent studies being significantly more likely to report the number of visits, transportation method, number of observers, and monitoring frequency. This suggests a professionalization of the field in Brazil, likely driven by the maturation of Road Ecology as a discipline. Conversely, our finding that larger number of authors are associated with higher omission of total distance monitored offers a provocative insight. We suggest that larger collaborations may suffer from fragmented data management, where foundational logistical metadata is lost during data synthesis. Additionally, the higher rates of reporting for infrastructure data (like concession status) in academic studies indicate that word count restrictions in article publishing may be incentivizing the removal of methodological details. To mitigate these omissions and ensure data replicability and comparability, we move beyond identifying gaps to proposing a Minimum Reporting Checklist (Table 1). This tool ensures that essential metadata is disclosed, either in the main text or as Supplementary Material, allowing future research to move from simple carcass counts to robust, comparable ecological inferences.

**Table 1:** Minimum Reporting Checklist (MRC) for roadkill studies in Road Ecology

Required variable (MRC checklist item)	Justification (why it is essential)
**Field survey and effort**	
Type of monitoring (e.g., vehicle, motorcycle, bus)	Crucial for comparing detectability and standardizing sampling effort.
Foot monitoring (if it was performed)	Crucial for comparing detectability and calculating corrected roadkill rates.
Vehicle speed (km/h)	A key factor influencing detection bias and replicability.
Number of observers	Necessary for calculating detection probability and observer effort.
Stretch length (km)	Defines the spatial scale of the study.
Total kilometers surveyed (km)	Essential input for calculating standardized roadkill rates.
Precise study duration	Needed to calculate total effort and estimate carcass persistence.
Number of visits	Needed to calculate total effort and estimate carcass persistence.
Monitoring frequency (e.g., monthly, weekly)	Important for estimating carcass persistence and removal rates, and essential for standardizing the temporal effort across studies.
**Infrastructure and management**	
Pavement type (paved/unpaved)	An important predictor of vehicle speed, traffic type, and mortality risk.
Number of lanes	An important predictor of vehicle speed, traffic type, and mortality risk.
Jurisdiction type (federal, state, municipal)	Relevant for policy application and governance structure.
Traffic volume	An important predictor of mortality risk and behavioral disturbance.
Road concession status	Determines responsibility for maintenance and mitigation funding, concessionaires usually survey the road more frequently.
**Context and mitigation**	
Presence and type of wildlife crossings (if any)	Essential for assessing the effectiveness of mitigation measures.
Proximity to protected areas	Necessary for assessing collision risk in conservation priority areas.
**Biological metrics**	
Number of species and individuals per taxon	Fundamental biodiversity and abundance metrics.
Species Accumulation Curve	Essential to validate the reported species richness and determine if the sampling effort was sufficient to capture local diversity.
Raw and corrected roadkill rate (individuals^.^km^–1.^day^–1^)	Allows for true impact assessment and corrects for carcass persistence/detection bias.
Threatened species status and proportion	Essential for identifying conservation priorities, assessing local extinction risks, and directing legally mandated mitigation measures toward endangered fauna.

Our MRC provides the precise methodological refinement currently lacking in standard environmental impact assessments for highway regularization and expansion. Federal guidelines already require seasonal roadkill censuses, mandating monthly campaigns across the entire road, early morning starts to reduce carcass removal bias, and a combination of vehicular and foot surveys (IBAMA, 2013; Ministério do Meio Ambiente, 2013; Ministério da Infraestrutura, 2020). These assessments are legally required to document survey effort, methodologies, species richness and abundance, hotspots, and mitigation proposals. By integrating the MRC into environmental licensing processes and ensuring that the resulting data are made publicly available, it will be possible to establish a standardized and robust database. This integration would bridge the gap between regulatory compliance and scientific research, enabling researchers to more reliably estimate roadkill rates, model the underlying risk factors, analyze spatiotemporal patterns, and accurately assess the impacts of roads on biodiversity.

### Roadkill Patterns: Richness, Abundance and Rates

The analysis revealed a taxonomic sampling bias in roadkill monitoring, notably impacting amphibians and reptiles. Despite Brazil’s status as the global leader in amphibian richness (Segalla et al., 2021) and the third most speciose when it comes to reptiles (Costa et al., 2021), these groups exhibited the lowest mean species richness, abundance, and collision rates. When appropriate methods are employed, amphibians can account for up to 90% of all road mortality (Kindel et al., 2024). The observed low metrics are not indicative of low impact but rather of a significant methodological bias. This is driven by the low persistence of small carcasses, with less than 60% persistence for larger amphibians (~180 g) within the first 24 hours (Barrientos et al., 2018; Santos et al., 2016). This issue is exacerbated by the known inefficiency of vehicle-based surveys in detecting small-bodied animals (Santos et al., 2016; Teixeira et al., 2013). Another reason for the underestimation of these groups may be related to temperature and seasonality, as their roadkill rates typically peak during warmer months (Carvalho-Roel et al., 2023; Pereira et al., 2025), probably due to concentrated reproductive activity (Pereira et al., 2018), and short-term studies may fail to encompass these critical periods. Consequently, current roadkill data severely underestimates collision mortality for these vulnerable groups, fundamentally undermining conservation efforts (Kindel et al., 2024).

Birds were among the most diverse and frequently road-killed. This is consistent with their high mobility, foraging behavior (attraction to grains, insects, and carrion), and specific low-altitude flight patterns, which heighten their exposure to collisions (Kociolek et al., 2015). Although carcass persistence for birds is longer than for amphibians, it still affects recording frequency (Barrientos et al., 2018), suggesting that current rates are still underestimated and not fully reflective of the impact on Brazil’s third-highest global bird endemism (Pacheco et al., 2021).

Mammals consistently ranked among the most abundant groups, and with the highest roadkill rates, reflecting their higher carcass persistence (Barrientos et al., 2018). While they represent a critical conservation concern (27% of species threatened) (IUCN, 2025), their high detectability often makes them the focus of roadkill research. Although most reviewed studies did not explicitly differentiate roadkill rates by body mass, most mammalian data in the literature regard medium and large-sized species. For instance, in a comprehensive Brazilian dataset comprising 7553 mammal roadkill records (identified to the species level), less than 4% of the entries belonged to small mammals, even though they account for 47% of the species identified as roadkill (Grilo et al., 2018). Therefore, small mammals are underestimated in standard surveys. This disproportionate gap within the same taxonomic class reinforces that the apparent dominance of mammals in roadkill statistics is size-dependent, masking a sampling deficit for small-bodied taxa. Critically, none of the roadkill rates presented here or in the current literature assessed reflect the true value of collision mortality because nearly all studies failed to apply correction factors for detection and carcass removal biases. This data limitation is significant because accurate, corrected roadkill rates are fundamental for assessing the direct impacts of roads, justifying mitigation costs, and defining priority segments for allocating public or private resources (Pinto et al., 2022). Therefore, the data reviewed serves as a minimum observed baseline, emphasizing the urgent need for a shift towards standardized, bias-corrected monitoring protocols across the national road network.

### Limitations

The primary limitation of this review stems from the methodological flaws of the primary studies. Our analysis was necessarily restricted to the observed (raw) roadkill rates reported in the literature, as less than 5% of the reviewed studies included foot surveys to account for carcass detection errors. This reliance on raw data means that all reported rates, including the differences quantified between taxonomic groups, reflect only a minimum baseline of impact. Consequently, our results likely underestimate the true collision mortality rates for all taxa, particularly for small-bodied animals, where the detection bias is known to be most severe.

While we included both peer-reviewed articles and gray literature (theses, dissertations) to maximize coverage, the inherent publication bias remains; studies with statistically significant or novel results may be more likely to be published or easier to locate. Furthermore, the reliance on regional gray literature, while essential for comprehensive coverage, introduces potential variability in the rigor of peer review compared to international journals.

## Conclusions

This systematic review confirms that while roadkill research in the Brazilian Cerrado has expanded significantly, the field is characterized by methodological inconsistencies and data gaps. As the first comprehensive evaluation of data omission in Brazil, this study demonstrates that these gaps create a fragmented evidence base that fundamentally compromises the effectiveness of environmental licensing and the planning of mitigation strategies.

Our analysis reveals that the systematic underestimation of small-bodied taxa is not a biological pattern, but a direct consequence of current monitoring protocols: a reliance on high-speed vehicle surveys, the scarcity of foot-based monitoring, and survey frequencies that do not account for rapid carcass removal. These methodological biases, coupled with a geographic concentration of research in socio-economically established states, leave critical agricultural frontiers, such as the Matopiba region, severely understudied despite their high deforestation rates and high ecological vulnerability to roads.

To transition roadkill monitoring from theoretical findings to applied ecological science, we propose two shifts. First, the adoption of a Minimum Reporting Checklist (MRC) must become standard practice, not only in academia but also as a requirement for regulatory agencies to ensure data comparability. Importantly, while our framework was developed within the Cerrado context, the parameters included in the MRC are fundamental to any roadkill survey; thus, it is designed to be universally applicable to monitoring programs worldwide, helping researchers report essential information for future meta-analyses. Second, research funding and environmental monitoring must be strategically redirected toward the Cerrado’s neglected frontiers. By addressing these reporting failures, we can ensure that decades of Road Ecology research are finally translated into evidence-based conservation actions that are as robust as the ecosystems they aim to protect.

## Supplementary information

Supplementary tables - Two decades of roadkill studies

## Data Availability

Data will be made available on request.
