# Supplementary material for: Bridging the Gap Between Theory and Practice: Assessing Two Decades of Methodological Procedures and Reporting Omissions in Roadkill Studies of The Brazilian Cerrado
Source: Environ Manage. 2026 Aug 17;76(9):274. doi: 10.1007/s00267-026-02564-4 (PMC13481585; doi:10.1007/s00267-026-02564-4)
Supplement: Supplementary file 1 [file 267_2026_2564_MOESM1_ESM.pdf]

Environmental Management

Bridging the gap between theory and practice: Assessing two decades of methodological procedures and reporting omissions in roadkill studies of the Brazilian Cerrado

Natalia de Quadros Pereira[1], Carine Firmino Carvalho-Roel[1,2*]

[1] Laboratório de Ecologia de Mamíferos (LEMA), Instituto de Biologia, Universidade Federal de Uberlândia, 38405-302, Rua Ceará (esquina com rua Amazonas), s/n, Bairro Umuarama, Uberlândia, MG, Brasil. nataliaqdros21@gmail.com. carinefcarvalho@gmail.com
[2] Laboratório de Ecologia e Conservação (LAEC), Departamento de Biologia, Faculdade de Filosofia, Ciências e Letras de Ribeirão Preto, Universidade de São Paulo (FFCLRP/USP), 14025-300, Rua Moreira de Oliveira, 354, Ribeirão Preto, São Paulo, Brasil.

[*] Corresponding author.
E-mail address: carinefcarvalho@gmail.com

Table S1 List of studies included in the systematic review and final analysis (n = 63)

Title	Year	Authors
A influência dos fatores da paisagem no atropelamento da fauna na GO-020 e GO-536, trecho entre Goiânia e Senador Canedo, estado de Goiás.	2020	Max Moraes Carneiro
Análise dos atropelamentos de mamíferos em uma rodovia no estado de São Paulo utilizando Self-Organizing Maps	2018	Larissa Sayuri Tsuda
Análise dos impactos ambientais dos atropelamentos de fauna na unidade de conservação do Parque Nacional da Chapada dos veadeiros – um estudo de caso na GO-239.	2022	Luana Pereira De Azevedo Lima
Anurofauna atropelada em rodovia no sul de minas gerais	2021	Caroline Scarabelin, Gabriela De S. Cavalcante, Luiz F. Ferreira, Paulo O. Garcia
Aspectos ecológicos e espaciais da fauna silvestre atropelada na APA Pouso Alto, Chapada dos Veadeiros	2018	Leonardo Pereira Fraga
Atropelamento de animais silvestres na rodovia go-060 entre Iporá e Arenópolis, estado de Goiás	2021	Jefferson Eduardo Silveira Miranda, Adryhano Dos Santos, Wellington Fernandes De Souza, Daniel Blamires
Atropelamento de vertebrados silvestres em uma região fragmentada do nordeste do estado de São Paulo: quantificação do impacto e análise de atores envolvidos.	2004	Cristina De Santis Prada
Atropelamentos de vertebrados nas rodovias MG-428 e SP-334 com análise dos fatores condicionantes e valoração econômica da fauna.	2009	Carlos Henrique De Freitas
Atropelamentos de vertebrados silvestres em quatro rodovias no Cerrado, Mato Grosso, Brasil	2018	Martins Valadão, Lilian Freitas Bastos, Carolina Pötter De Castro
Atropelamentos e influência da paisagem na sobrevivência de mamíferos silvestres de médio e grande porte	2018	Juliane Fernandes Guimaraes, Claudionor Ribeiro Da Silva, Marco Aurélio Alves Perin
Avaliação do uso de controladores eletrônicos de velocidade como medida de mitigação de atropelamentos de animais silvestres na rodovia BR-262, trecho de Anastácio à Corumbá, Mato Grosso do Sul	2016	Marcela Barcelos Sobanski
Como as características da paisagem e rodovia explicam atropelamentos em áreas protegidas à escala local?	2016	Nayara Helena Alecrim De Freitas
Comparação histórica dos atropelamentos de animais silvestres na rodovia MG-428	2017	Lerrane De Fatima Cunha

Composição e história natural das serpentes de Cerrado de Itirapina, São Paulo, sudeste do Brasil	2008	Ricardo Jannini Sawaya, Otavio Augusto Vuolo Marques & Marcio Martins
Dos Impactos à Conservação da Fauna: a implantação do campus Lagoa do Sino e a incidência de atropelamentos de animais silvestres	2018	Adriana Paula Bernardo Cravo
Efeito da paisagem sobre os atropelamentos de mamíferos de médio e grande porte no sul de Goiás, Brasil	2020	Larissa Aparecida De Freitas Araujo, Wellington Hannibal, Raoni Ribeiro Guedes Fonseca Costa, Reile Ferreira Rossi, Hermes Willyan Parreira Claro
Efeitos da BR-070 na Província Serrana de Cáceres, Mato Grosso, sobre a comunidade de vertebrados silvestres	2007	Elisabete Segatto Melo & Manoel Santos-Filho
Estudo das espécies vítimas de atropelamento na rodovia BR-262, no trecho Uberaba–Peirópolis (Triângulo Mineiro, MG, Brasil)	2014	Cristiane M. Santos, Pedro H. M. Fonseca, Thiago S. Marinho, Gabriel C. Cunha, Edson A. Santos, Maria H. Soares, Camila L. Cavellani, Mara L. F. Ferraz, Ana E. Iannini Custódio, Vicente P. A.Teixeira, Agustín G. Martinelli
Evolução dos índices de atropelamento de vertebrados silvestres nas rodovias do entorno da Estação Ecológica Águas Emendadas, DF, Brasil, e eficácia de medidas mitigadoras	2006	Tathiana Bagatini
Fast and furious: a look at the death of animals on the highway MS-080, Southwestern Brazil	2014	Naira C. De Carvalho, Marcelo O. Bordignon, Julie T. Shapiro
Fatores potencializadores dos atropelamentos de fauna silvestre no trecho da GO-080 entre Goiânia e Petrolina de Goiás	2024	Helena Bernardes Cortez, Fabrizia Gioppo Nunes, Alex Mota Dos Santos
Fauna atropelada nas estradas do município de Chapadão do Céu (Goiás, Brasil)	2015	Carlos de Melo e Silva Neto, Vandervilson Alves Carneiro, Bruno Bastos Gonçalves, Fernando José Da Cruz Ribeiro
Fauna silvestre atropelada na br-135: avaliação do padrão espacial dos atropelamentos e análise de fatores ecológicos preditivos	2022	Marina De Souza
Genética aplicada à ecologia de estradas: um estudo na estrada municipal Guilherme Scatena (São Carlos, SP) e potencial impacto na biodiversidade local.	2012	Karen Giselle Rodrígez Castro
Hotspot analysis of wildlife vehicle collisions of BR-262, Mato Grosso do Sul, Brazil and recommendations for mitigation efforts	2015	Rebecca R. Dougherty
Hotspots and Season Related to Wildlife Roadkill in the Amazonia–Cerrado Transition	2022	Evandro Santos, Milton Cordova, Clarissa Rosa, Domingos Rodrigues

Impacto das rodovias sobre a fauna silvestre: levantamento do índice de atropelamento de vertebrados nas rodovias do entorno da Estação Ecológica Águas Emendadas	2016	Percilia Margarete Moitinho Souza
Impactos das estradas na fauna silvestre – dinâmica de atropelamentos de vertebrados em trechos de duas rodovias do centro-oeste de Minas Gerais	2022	Lucas Antônio Marques, Débora Nogueira Campos Lobato, Júnio Damasceno de Souza, Camila Mariangela Pacheco
Impactos de atropelamentos de animais silvestres no trecho da rodovia SP–215 confrontante ao Parque Estadual de Porto Ferreira – Porto Ferreira, SP	2010	Sonia Aparecida de Souza, André Luis Teixeira de Lucca, Ernesto Pedro Dickfeldt, Paulo Roberto de Oliveira
Influência dos fatores naturais e antrópicos no atropelamento de mamíferos na BR-163, Mato Grosso.	2021	Luís Renato Rezende Bernardo
Influências da pavimentação de rodovias em índices de atropelamento de fauna: o caso da rodovia GO-239 em Alto Paraíso de Goiás.	2016	Tatiana Rolim Soares Ribeiro
Levantamento das espécies vítimas de atropelamento em um trecho da rodovia BR-262, trajeto Uberaba-Peirópolis-Ponte Alta (MG)	2014	Santos, C.M. Martinelli, A.G., Fonseca, P.H.M. , Cardoso, G.C. , Soares, M.H. , Santos, E.A. , Cavellani, C.L., Teixeira, V.P.A. , Ferraz, M.L.F.
Levantamento de animais silvestres atropelados em rodovias da região de Ribeirão Preto-SP nos anos de 2004 a 2006	2010	Andrés Ricardo Domingos
Levantamento de animais vertebrados atropelados em trechos das rodovias MG-352 e MG-190	2020	Leonardo Machado da Silva, Francielle Aparecida de Sousa, Thays Cunha Vieira, Cássio Resende de Morais
Levantamento de animais vertebrados vítimas de atropelamentos em trechos das rodovias MG-223, MG-190 e BRr-352	2017	Pablo Anderson Silva de Oliveira, Eduardo de Freitas Sousa, Francine Borges Silva
Levantamento de fauna atropelada na GO 060, trecho Trindade - Santa Bárbara de Goiás	2024	Bruna Paula da Silva Cunha, Maria Eduarda Gomes Dos Santos, Nathalya Pinheiro Dos Santos, Susy Ricardo Lemes Pontes
Levantamento de vertebrados silvestres atropelados no trecho rodoviário de Itapuranga a Ceres, Goiás	2019	Diego André Barbosa Cesário, Laís Naiara Gonçalves dos Reis
Levantamento de vertebrados silvestres atropelados com enfoque em indivíduos da ordem Chiroptera: estudo de caso da rodovia MGC-354, Minas Gerais, Brasil	2015	Daniel Magella Damasceno Alves, Rafael Ferraz de Barros, Consuelo Nepomuceno
Matriz de entorno de rodovia e ocorrência de atropelamentos influenciam a abundância de *Caracara*	2019	Daniela Caixeta Oliveira

plancus (Aves: Falconiformes) e *Coragyps atratus* (Aves: Cathartiformes)?		
Monitoramento de animais silvestres atropelados na rodovia MGC-455, trecho Uberlândia-Rio do Peixe, Uberlândia-MG.	2015	Wanessa Gomes Pereira
Mortalidade de vertebrados por atropelamento em um trecho da GO–164, no sudoeste Goiano	2013	Raoni Ribeiro Guedes Fonseca Costa, Líllian Andrade Dias
O impacto das rodovias sobre a fauna de vertebrados silvestres no cerrado mineiro	2011	Robinson Moiséis da Silva, Cristiano Humberto Orsine Borba, Vivian Prado de Camargo Leão, Marina Farcic Mineo
O método de amostragem e as características físicas da rodovia MGC-497, trecho Uberlândia-Prata, MG, influenciam as taxas de atropelamento da fauna silvestre?	2023	Nayane Alves Da Silva
Padrões espaciais e temporais no atropelamento de vertebrados silvestres em duas Estradas-Parque no Cerrado do Brasil Central.	2019	Samara Maciel
Quantificação de atropelamentos de vertebrados silvestres em um trecho da GO 213	2011	Kaio César Damacena Silva, Dhesy Allax Cândido de Freitas, Kyrly Morais Carneiro, Nilson Kássio Pereira Lima, Pâmella Aparecida de Souza Pires, Jonas Byk
Registro de atropelamento de animais silvestres entre as cidades de Palmeiras de Goiás e Edealina –GO	2013	Diogo Cordeiro Gomes, Cláudia Valéria da Silva, Anderson Assis de Faria, Marco Antônio Vieira Morais, Carlos Eduardo Ramos Sant'Ana, Luiz Gonzaga Alves Mendonça
Resultados preliminares do subprograma de controle de atropelamento de fauna BR-060.	2012	Heider Damas Vieira, Samuel Bernardes, Diego Tristão, Vivianne Velozo, Gisele Souza
Roadkill hotspots in a protected area of Cerrado in Brazil: planning actions to conservation	2016	Bruno H Saranholi, Mariano M. Bergel, Paulo HP Ruffino, Karen G Rodríguez-C, Lucas A Ramazzotto, Biotec, Patrícia D De Freitas, Pedro M Galetti Jr.
Roadkill in the Brazilian Cerrado savanna: comparing five highways in southwestern Goiás	2017	Jefferson Eduardo Silveira Miranda, Ricardo Keichi Umetsu, Fabiano Rodrigues de Melo, Fabiana Cristina Silveira Alves Melo, Kleber Fernando Pereira, Seixas Rezende Oliveira
Roadkill of wild vertebrates along the GO-060 road between Goiânia and Iporá, Goiás State, Brazil	2010	Hélida Ferreira Da Cunha, Fabiane Geralda Alves Moreira, Silvania de Sousa Silva

Roadkill on vertebrates in Brazil: seasonal variation and road type comparison	2015	Felipe S. Machado, Marco A. Fontes, Pedro B. Mendes, Aloysio S. Moura, Beatriz dos S. Romão
Roadkill records of reptiles and birds in Cerrado and Pantanal landscapes	2018	Wagner Fischer, Raquel Faria Godoi, Antonio Conceição Paranhos Filho
Spatial and temporal influences of road duplication on wildlife road kill using habitat suitability models	2014	Giordano Ciocheti
Spatial patterns of road mortality of medium–large mammals in Mato Grosso do Sul, Brazil	2017	Fernando Ascensão, Arnaud LJ Desbiez, Emília P. Médici, Alex Bager
Telemetria convencional e via satélite na determinação da área de vida de três espécies de carnívoros da região nordeste do estado de São Paulo	2001	José Eduardo Mantovani
Tendências temporais nos atropelamentos de mamíferos de médio e grande porte em um trecho de rodovia no sudeste do Brasil	2022	Ana Flávia Pereira Domiciano
The influence of proximity with riparian forests and the distance from urban areas on roadkills of vertebrates in a fragmented Brazilian savanna area	2023	Thais Martins, Simone Rodrigues Freitas, Décio Luis Semensatto Jr, Elisa Hardt
The spatial, climatic and temporal factors influencing roadkill change according to the taxonomic level	2023	Carine Firmino Carvalho-Roel, Ana Elizabeth Iannini Custódio, Oswaldo Marçal Júnior, Clara Grilo
Variação da taxa de atropelamento de animais entre rodovias com diferentes tipos de pavimentação e número de pistas.	2013	Almir Picanço de Figueiredo, Rodrigo Augusto Dos Santos Lima, Caroline de Mello Soares
Variação espacial e sazonal de atropelamentos de mamíferos no Bioma Cerrado, rodovia BR 262, sudoeste do Brasil	2012	Nilton C. Cáceres, Janaina Casella, Charla Dos Santos Goulart
Variação sazonal da fauna selvagem atropelada na rodovia MG 354, sul de Minas Gerais – Brasil	2012	André Luis Pracucci Gomes dos Santos, Clarissa Alves da Rosa, Alex Bager
Wild vertebrate roadkill in the Chapada dos Veadeiros National Park, Central Brazil	2016	Vívian Da Silva Braz & Frederico Gustavo Rodrigues França
Wildlife roadkill in the surroundings of Emas National Park, Cerrado biome, Brazil	2021	Carine Firmino Carvalho-Roel, Giselle Bastos Alves, Anah Tereza de Almeida Jácomo, Renato Alves Moreira, Natália Mundim Tôrres, Leandro Silveira

Table S2 Studies recorded in the Cerrado domain but excluded from the systematic review due to inclusion or exclusion criteria (n = 45)

Study title	Dissemination type	Reason for exclusion
A fauna atropelada em rodovias do sudoeste goiano e as implicações para a conservação	Master's theses	Data from a computed database
Ameaças à sobrevivência de animais silvestres no estado de Goiás	Article	Main focus was not related to roadkill
An estimate of wild mammal roadkill in São Paulo state, Brazil	Article	Covered another biome
Are roadkill hotspots in the Cerrado equal among groups of vertebrates?	Article	Data from a computed database
Assessing the consistency of hotspot and hot-moment patterns of wildlife road mortality over time	Article	Data from a computed database
Atropelamentos de tatu-canastra *Priodontes maximus* (Kerr, 1792) em uma rodovia no cerrado goiano e sua relação com a paisagem do entorno	Article	Covered only one species
Biologia e conservação do lobo-guará na estação ecológica de águas emendadas, DF	Doctoral dissertation	Main focus was not related to roadkill
Caracterização da fauna helmintológica de tamanduá-bandeira (*Myrmecophaga tridactyla*) e tamanduá-mirim (*Tamandua tetradactyla*) atropelados nas rodovias BR-050 e BR-455 (Minas Gerais, Brasil)	Article	Data from a computed database
Como a velocidade de avaliação interfere no número de registro de animais atropelados?	Article	Incomplete data
Dinâmica das rodovias: o papel do tráfego nos índices de atropelamentos de fauna	Article	No species list
Dinâmica de atropelamento de fauna silvestre no entorno de unidades de conservação do Distrito Federal	Doctoral dissertation	Data from a computed database
Diversidade e genética de *Euphractus sexcinctus* (Linnaeus, 1758) em áreas de cerrado e pantanal	Doctoral dissertation	Covered only one species
Diversidade genética e conservação do lobo-guará, *Chrysocyon brachyurus*, em áreas protegidas do Distrito Federal	Master's theses	Main focus was not related to roadkill
Ecologia de estradas: a importância do tempo de estudo na localização dos hotspots para herpetofauna (Reptilia)	Undergraduate theses	Incomplete data
Efeito da implantação de rodovias no Cerrado brasileiro sobre a fauna de vertebrados	Article	Covered another biome
Effects of roads on the vertebrates diversity of the indigenous territory Paresi and its surrounding	Article	Incomplete data
Estudos preliminares da presença de sagüis no município de Bauru, São Paulo, Brasil	Article	Main focus was not related to roadkill
Fatores ecológicos que influenciam a agregação de territórios em tizius (*Volatinia jacarina*) e sua relação com o risco de atropelamento	Undergraduate theses	Main focus was not related to roadkill
Hotspots de atropelamentos e a influência da paisagem na sobrevivência de mamíferos de médio e grande porte em uma área do Cerrado mineiro	Master's theses	Data from a computed database
How landscape features influence road-kill of three species of mammals in the Brazilian savanna?	Article	No species list

Identification of microsatellite DNA markers for the giant anteater *Myrmecophaga tridactyla*	Article	Main focus was not related to roadkill
Impacto do atropelamento de animais silvestres na rodovia GO 060, trecho Goiânia - Iporá.	Master's theses	Data from a computed database
Impacto do atropelamento em rodovias sobre a fauna do cerrado	Master's theses	Literature Review
Influence of climate variables on roadkill rates of wild vertebrates in the Cerrado biome, Brazil	Article	Data from a computed database
Levantamento da mastofauna e identificação das influências antrópicas em dois fragmentos de Mata Atlântica transição com Cerrado de Rubião Júnior – Botucatu - SP	Undergraduate theses	Main focus was not related to roadkill
Levantamento de animais silvestres atropelados em trecho da rodovia BR482	Article	Biome not specified
Levantamento de fauna atropelada na GO 050, trecho Trindade - Campestre de Goiás	Article	Incomplete data
Levantamento de vertebrados silvestres atropelados no trecho rodoviário de Itapuranga a Ceres, Goiás	Article	Data from a computed database
Mamíferos do cerrado de mato grosso, com ênfase no uso do espaço por *Cerdocyon thous* (Carnivora, Canidae) e *Mazama gouazoubira* (Artyodctyla, Cervidae).	Doctoral dissertation	Main focus was not related to roadkill
Mammal occurrence and roadkill in two adjacent ecoregions (Atlantic Forest and Cerrado) in South-western Brazil	Article	Covered another biome
Mammal road mortality and cost–benefit analyses of mitigation measures aimed at reducing collisions with capybara (*Hydrochoerus hydrochaeris*) in São Paulo state, Brazil	Article	Biome not specified
Monitoramento de animais silvestres atropelados em um trecho de mata fragmentado pela BR-153/GO-060	Article	Data from a computed database
Padrões espaciais e temporais no atropelamento de vertebrados silvestres em duas estradas-parque no Cerrado do Brasil central.	Master's theses	Data from a computed database
Padrões espacial e temporal do atropelamento de mamíferos em uma rodovia no cerrado brasileiro	Master's theses	Data from a computed database
Predicting spatiotemporal patterns of road mortality for medium-large mammals	Article	Incomplete data
Relação entre atropelamentos de fauna e o volume de visitações no Parque Nacional da Chapada dos Veadeiros: o caso da rodovia GO-239	Article	Data from a computed database
Roadkill on vertebrates in Brazil: seasonal variation and road type comparison	Article	Biome not specified
SIG de animais silvestres atropelados no centro goiano	Article	Incomplete data
Spatial and temporal influences of road duplication on wildlife road kill using habitat suitability models	Doctoral dissertation	Data from a computed database
Taxocenoses de serpentes em grupos fitofisionômicos de Cerrado no Parque Nacional de Sete Cidades, Piracuruca, Piauí, Brasil	Master's theses	Main focus was not related to roadkill
The effect of roadkills on the persistence of xenarthran populations: the case of the Brazilian Cerrado	Article	Literature Review

Threats to and viability of the giant anteater, *Myrmecophaga tridactyla* (Pilosa: Myrmecophagidae), in a protected Cerrado remnant encroached by urban expansion in central Brazil	Article	Main focus was not related to roadkill
Using DNA barcode to relate landscape attributes to small vertebrate roadkill	Article	Data from a computed database
Uso de sensoriamento remoto e análise espacial na interpretação de atropelamentos de fauna entre Campo Grande e Aquidauana, MS	Conference proceedings	Data from a computed database
Vertebrate roadkill: identifying where, when and who dies on wildlife vehicle collisions on Brazilian Cerrado	Doctoral dissertation	Data from a computed database

Table S3 Scientific journals (n=30) that published the primary studies included in the systematic review

Scientific journal name	Number of studies
Oecologia Australis	3
Biota Neotropica	2
Revista Brasileira de Zoociências	2
Revista GeTeC	2
Journal of Environmental Management	1
Revista Enciclopédia Biosfera	1
Acta Scientiarum. Biological sciences	1
Austral Ecology	1
Brazilian Journal of Development	1
Check List	1
Diversity	1
Enciclopédia Biosfera	1
Historia Natural	1
Iheringia. Série Zoologia	1
Mastozoologia Neotropical	1
Multi-Science Jornal	1
North-Western Journal Of Zoology	1
Revista Caminhos De Geografia	1
Revista Ceciliana	1
Revista de Biotecnologia & Ciência	1
Revista do Instituto Florestal	1
Revista Eletrônica Interdisciplinar - REI	1
Revista Heringeriana	1
Revista Ibero-Americana de Ciências Ambientais	1
Revista M Córdoba	1
Revista Percurso	1
Revista Perquirere	1
Vita e Sanitas	1
Wildlife Research	1

Table S4 Definitions of methodological variables assessed during the review of studies about roadkill performed in Brazilian Cerrado (2000-2024)

Number of visits: The total count of sampling occasions or monitoring events conducted throughout the study period.
Road stretch: The specific linear extent or segment of the road network selected for the study (e.g., a specific 50-km section between two cities).
Total distance monitored: The cumulative linear distance (e.g., kilometers) covered during the road surveys (calculated as the length of the road stretch multiplied by the number of visits).
Data collection duration: The total temporal span of the field activities, measured as the number of months between the start and end of data collection.
Traffic volume: Information regarding vehicular flow or traffic density on the studied road stretch.
Pavement type: Characterization of the road surface material, typically categorized as paved (asphalt/concrete) or unpaved (dirt/gravel).
Road lane configuration: Description of the road structure regarding the number of lanes (e.g., two-lane, four-lane).
Concession status: Identification of whether the road is managed by a private concessionaire or by a public agency.
Road jurisdiction type: Administrative level responsible for the road (municipal, state, federal).
Transportation method: The specific mode of transport utilized by researchers during monitoring (e.g., automobile, motorcycle, bicycle, or other).
Vehicle speed: The average speed maintained by the vehicle during the road surveys.
Number of observers: The total number of researchers or observers actively participating in the data collection process during surveys.
Monitoring frequency: The periodicity of the surveys (e.g., daily, weekly, monthly, or bimonthly).
PA: If the road passes through a protected area, as reported in the study.

Table S5 Analysis of Deviance (Type II Likelihood Ratio Chi-square tests) for the influence of bibliometric and temporal drivers on the omitting of methodological parameters in Brazilian Cerrado roadkill studies (n = 63) (2000-2024)

Number of visits			
Term	χ^2	df	p
Year	6.591	1.000	**0.010**
Authors	1.155	1.000	0.282
Language	0.488	1.000	0.485
Dissemination_type	1.843	2.000	0.398
**Total distance monitored**			
Term	χ^2	df	p
Year	0.858	1.000	0.354
Dissemination_type	2.051	2.000	0.359
Authors	4.635	1.000	**0.031**
Language	1.363	1.000	0.243
**Traffic volume**			
Term	χ^2	df	p
Year	0.168	1.000	0.682
Dissemination_type	4.534	2.000	0.104
Authors	2.266	1.000	0.132
Language	1.014	1.000	0.314
**Road lane configuration**			
Term	χ^2	df	p
Year	0.009	1.000	0.925
Dissemination_type	4.345	2.000	0.114
Authors	0.085	1.000	0.770
Language	0.923	1.000	0.337
**Concession status**			
Term	χ^2	df	p
Year	0.027	1.000	0.869
Dissemination_type	8.097	2.000	**0.017**
Authors	2.515	1.000	0.113
Language	0.151	1.000	0.698
**Transportation method**			
Term	χ^2	df	p
Year	16.736	1.000	**0.000**
Dissemination_type	0.520	2.000	0.771
Authors	0.247	1.000	0.620
Language	2.710	1.000	0.100
**Vehicle speed**			
Term	χ^2	df	p
Year	2.620	1.000	0.106
Dissemination_type	0.452	2.000	0.798
Authors	0.325	1.000	0.569
Language	0.495	1.000	0.482

Number of observers			
Term	χ^2	df	p
Year	3.716	1.000	**0.054**
Dissemination_type	1.616	2.000	0.446
Authors	2.339	1.000	0.126
Language	1.091	1.000	0.296
Monitoring frequency			
Term	χ^2	df	p
Year	4.423	1.000	**0.035**
Dissemination_type	1.106	2.000	0.575
Authors	0.034	1.000	0.854
Language	0.310	1.000	0.578
Omission score			
Term	χ^2	df	p
Year	15.134	1.000	**0.000**
Dissemination_type	0.519	2.000	0.771
Authors	0.020	1.000	0.888
Language	0.497	1.000	0.481

Omission score: A cumulative index representing the total number of unreported items across the 12 assessed methodological variables (ranging from 0 to 12).

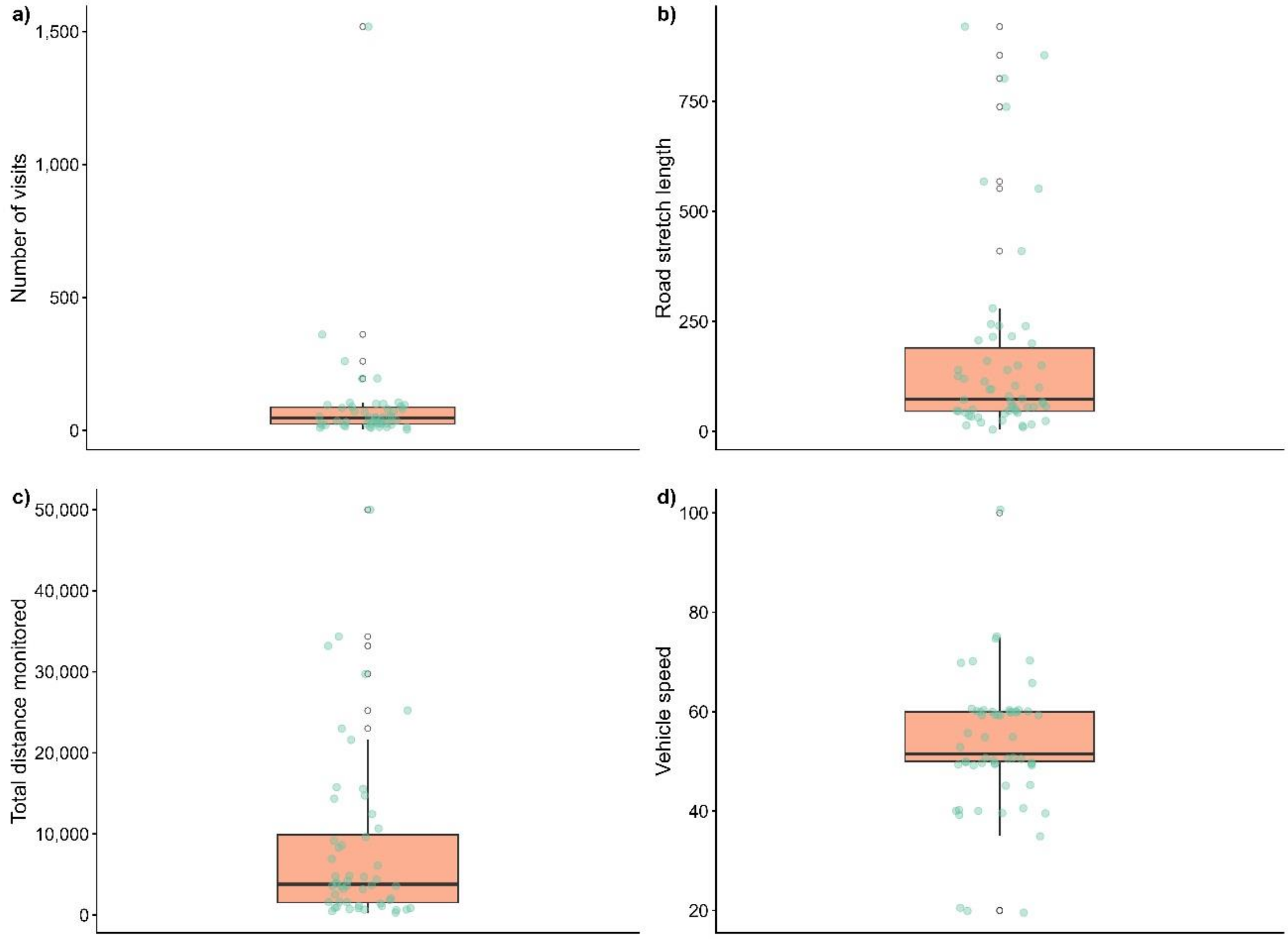

Fig. S1 Variability in methodological survey effort across 63 roadkill studies performed in Brazilian Cerrado (2000-2024): a) number of visits, b) road stretch length (km), c) total distance monitored (km), and d) vehicle speed (km/h)
